# Serum insulin-like growth factor binding protein 3 as a promising diagnostic and prognostic biomarker in esophagogastric junction adenocarcinoma

**DOI:** 10.1007/s12672-022-00591-1

**Published:** 2022-11-21

**Authors:** Tian-Yan Ding, Yu-Hui Peng, Chao-Qun Hong, Bin-Liang Huang, Can-Tong Liu, Yun Luo, Ling-Yu Chu, Biao Zhang, Xin-Hao Li, Qi-Qi Qu, Yi-Wei Xu, Fang-Cai Wu

**Affiliations:** 1 Department of Clinical Laboratory Medicine, The Cancer Hospital of Shantou University Medical College, Shantou, 515041 Guangdong China; 2 Esophageal Cancer Prevention and Control Research Center, The Cancer Hospital of Shantou University Medical College, Shantou, 515041 Guangdong China; 3 Guangdong Esophageal Cancer Institute, Guangzhou, 510000 Guangdong China; 4 Department of Oncological Laboratory Research, The Cancer Hospital of Shantou University Medical College, Shantou, 515041 Guangdong China; 5 Department of Radiation Oncology, The Cancer Hospital of Shantou University Medical College, Shantou, 515041 Guangdong China

**Keywords:** IGFBP3, Nomogram, Diagnosis, Prognosis, Esophagogastric junction adenocarcinoma

## Abstract

**Background:**

Esophagogastric junction adenocarcinoma (EJA) lacks serum biomarkers to assist in diagnosis and prognosis. Here, we aimed to evaluate the diagnostic and prognostic value of serum insulin-like growth factor binding protein 3 **(**IGFBP3) in EJA patients.

**Methods:**

320 participants were recruited from November 2016 to January 2020, who were randomly divided into a training cohort (112 normal controls and 102 EJA patients including 24 early-stage patients) and a validation cohort (56 normal controls and 50 EJA patients including 12 early-stage patients). We used receiver operating characteristics curve (ROC) to evaluate diagnostic value. The predictive performance of the nomogram was evaluated by the concordance index (C-index).

**Results:**

Serum IGFBP3 levels were significantly lower in early-stage EJA or EJA patients than those in controls (P < 0.01). Measurement of serum IGFBP3 demonstrated an area under curve of 0.819, specificity 90.18% and sensitivity 43.14% in training cohort. Similar results were observed in validation cohort (0.804, 87.50%, 42.00%). Importantly, serum IGFBP3 had a satisfactory diagnostic value for early-stage EJA (0.822, 90.18%, 45.83% and 0.811, 84.48%, 50.00% in training and validation cohorts, respectively). Furthermore, survival analysis demonstrated that lower serum IGFBP3 level was related to poor prognosis (P < 0.05). Cox multivariate analysis revealed that serum IGFBP3 was an independent prognostic factor (HR = 0.468, P = 0.005). Compared with TNM stage, a nomogram based on serum IGFBP3, tumor size and TNM stage indicated an improved C-index in prognostic prediction (0.625 vs. 0.735, P = 0.001).

**Conclusions:**

We found that serum IGFBP3 was a potential diagnostic and prognostic marker of EJA. Meanwhile, the nomogram might predict the prognosis of EJA more accurately and efficiently.

**Supplementary Information:**

The online version contains supplementary material available at 10.1007/s12672-022-00591-1.

## Introduction

In recent decades, esophagogastric junction adenocarcinoma (EJA) has attracted considerable attention because of its sharp increase incidence in the worldwide [1–3]. However, the definition of EJA is still a controversial topic. Siewert et al. introduced a classification that EJA is defined as cancer with the epicenter within 5 cm of the esophagogastric junction, and tumors located between 1 cm above and 2 cm below the esophagogastric junction are described as EJA type II or “true carcinoma of the cardia” [4]. Nishi et al. put forward another view that irrespective of the histological subtype, tumors within 2 cm (above or below) of the esophagogastric junction should be defined as EJA [5]. No matter whether the tumor epicenter is located in the proximal stomach or the distal esophagus, EJA originates from the epithelial tissue of the esophagogastric junction. Up to now, the consensus is that EJA has specific characteristics in epidemiology and prognosis, and it is an aggressive disease with early lymphatic and blood spread. At present, surgery is still the best curative treatment option, but it is accompanied by high morbidity and mortality [6]. Most patients treated by surgery relapsed within 2 years, and the 5-year survival rate rarely exceeded 40% [7, 8]. The onset of EJA is hidden, and most patients are already in an advanced stage at the time of diagnosis, which is the main reason for its low survival rate[9]. Thus, it would be desirable to find a safe, efficient and simple method to identify EJA at an early stage for improving the survival of EJA patients.

Although studies showed that early detection can effectively reduce cancer mortality and burden, the special pathophysiological differences of EJA made it a great challenge to find an efficient and acceptable early detection method [10]. Nowadays, the endoscopy used in the early diagnosis of EJA is not suitable for the screening of the asymptomatic population because of its invasiveness [11]. On the other hand, the prognostic assessment of EJA, especially before receiving treatment, is also of great concern. The current prognosis evaluation of EJA is mainly based on the TNM staging system of gastric cancer and esophageal cancer. EJA still lacks its own TNM staging system. It has been suggested that the development of reliable biomarkers that could be detected in blood, saliva or urine samples might be widely used in the early detection of cancer. In recent decades, increasing serum tumor biomarkers, such as miRNAs, metabolites and circulating tumor cells, have been found and further studied [12–14]. However, these biomarkers used for early cancer detection rarely surpassed blinded Phase III validation studies, and their clinical value was still controversial [15, 16]. It is believed that there is a long way to go regarding the tumor biomarkers to be used in clinical practice.

Insulin-like growth factor binding protein3 (IGFBP3) is one of the six structurally related proteins of the IGFBP family and is a major carrier protein for insulin-like growth factor (IGF)-1 and -2 in circulation binding more than 90% of IGF in the serum [17]. As a multifunctional protein, IGFBP3 can impact on several molecular mechanisms that regulate cell survival or apoptosis, especially in the context of cancer [18]. Many previous studies suggested that the alteration of IGFBP3 could affect many types of tumors including hepatocellular carcinoma [19], breast cancer [20], and lung cancer [21]. A study revealed that compared with healthy subjects, the level of serum IGFBP3 in patients with gastric cancer decreased significantly [22]. However, what the role of IGFBP3 is in EJA has not been reported. Whether serum IGFBP3 has diagnostic and prognostic potential for EJA is worthy to be studied. The purpose of our study is to judge the diagnostic and prognostic value of serum IGFBP3 for EJA. And we hoped to establish an effective prognostic nomogram to predict the overall survival (OS) of EJA patients, which might help provide personalized treatment suggestions for clinicians.

## Methods

### Study population

This study retrospectively collected data from patients diagnosed with EJA and healthy volunteers. They were recruited at the Cancer Hospital of Shantou University Medical College from November 2016 to January 2020. We reviewed the detailed medical records of these patients who were diagnosed based on spiral computed-tomography (CT) and endoscopic examination followed by histopathology. According to the results of the medical examinations, patients were tumor-node-metastasis (TNM) staged based on the 8th edition of the American Joint Committee on Cancer (AJCC) Cancer Staging Manual [23]. Tumors with AJCC stages I + II were defined as early-stage EJA.

Patients included in the analysis met the following criteria: (1) they were diagnosed as EJA with histopathological examination [23]; (2) they had no history of cancer in other organs until EJA diagnosis or did not receive any anti-cancer treatment; (3) they had complete baseline clinical information and follow-up time data. We excluded those patients who died of surgical complications. Excluding cases that did not meet the requirements, a total of 152 patients were enrolled according to the inclusion criteria (58 patients only received excision alone, 75 patients received postoperative adjuvant chemotherapy, and 19 patients received other treatments). Healthy volunteers, as normal controls, had no evidence of tumor in their physical examination, which was obtained from the physical examination center in the same hospital. All enrolled participants were randomly grouped into training and validation cohorts. The OS was defined as the interval between the initial diagnosis and either death of cancer or the last follow-up. This study was approved by the Ethics Committee in Shantou University Cancer Center. All participants in this study obtained informed consent prior to the use of serum samples. All work was complied with the principles of the Helsinki Declaration.

### Enzyme-linked immunosorbent assay (ELISA) for serumIGFBP3

Serum samples were obtained from the included patients prior to treatment. The IGFBP3 concentration in the serum was measured with a IGFBP3 ELISA Kit (CUSABIO, Wuhan, China). Reagents, samples and standards were prepared as instructed. 100 μl standards and serum samples (a 100-fold dilution) were added per well and incubated for 2 h at 37 ℃. Then the liquid was removed and 100 μl of biotin-antibody (1 ×) was added to each well, and the plate was incubated for 1 h at 37 ℃. After washing, 100 μl HRP-avidin was added and incubated for 1 h at 37 ℃, followed by the same washing procedure for another five times. 90 μl TMB substrate was added to each well, and the plate was incubated again for 20 min at 37 ℃ protected from light. Color formation was stopped by adding 50 μl Stop Solution. The optical density (OD) of each well was read at wavelength of 450 nm and 590 nm as reference on a plate microplate reader (Multiskan MK3, ThermoFisher Scientific, Boston, USA) within 5 min. Corresponding concentrations were converted from OD values using standard curve method. All ELISA assays were performed in duplicates. The optimal threshold concentrations of IGFBP3 for diagnosis were determined through the analysis of the working characteristic curve of the subjects.

### Demographics and clinicopathologic characteristics

The original data of relevant demographics and clinicopathologic features of patients with EJA were collected for each enrolled patient, and exported from hospital’s electronic medical records system. The TNM stage of each patient was restaged based on the 8th AJCC TNM staging system [23]. The following clinical pathological data were included in the study: age, gender, smoking, alcohol drinking, tumor size, tumor invasion depth, regional lymph nodes, TNM stage and IGFBP3.

### Nomogram construction and assessment

In this study, continuous variables were transformed into categorical variables by X-tile software [24]. We used univariate analysis to select useful prognostic variables (P ≤ 0.05), and then conducted multivariate Cox regression analysis. A dynamic predictive nomogram with endpoints of 1-, 3- and 5-year OS was constructed based on all variables with a P-value of less than 0.05 in a multivariate model. By comparing with selected prognostic variables and traditional TNM staging system, the discrimination and calibration of the nomogram were examined with concordance index (C-index) and calibration curve, respectively. We used Akaike Information Criterion (AIC) [25] and Bayesian Information Criterion (BIC) [26] to assess the new model’s goodness-of-fit. Net reclassification improvement (NRI) [27] and integrated discrimination improvement (IDI) [28] were used to show the improvement of the new model. We used decision curve analysis (DCA) [29] and clinical impact curve (CIC) to estimate the clinical utility of the prognostic nomogram.

### Bioinformatic analyses and DNA methylation of the IGFBP3 gene

A freely available interactive resource is presented as part of the Human Protein Atlas portal (https://www.proteinatlas.org), and we used it to explore the expression and prognostic value of IGFBP3 in related cancer tissues. We used MethSurv (https://biit.cs.ut.ee/methsurv/, accessed on 30 October 2022) to determine the expression and prognostic patterns of single CpG methylation of the IGFBP3 gene. In this analysis, DNA methylation values are represented using beta values (beta values ranging from 0 to 1).

### Statistical analyses

Statistical analyses were performed using SPSS software (version 26.0, IBM Corp., Chicago, IL, USA), GraphPad Prism software (version 8.0.2, http://www.graphpad.com) and R (version 4.2.1, http://www.R-project.org) for Windows. We used ANOVA to compare the difference of serum IGFBP3 between EJA patients, early-stage EJA patients and normal controls. The diagnostic parameters of IGFBP3 were assessed by receiver operating characteristic (ROC) curve. The best cut-off value of serum IGFBP3 in diagnosing EJA was evaluated by achieving the maximum sensitivity when the specificity was > 90% [30]. The survival rate and significant difference were calculated by the Kaplan–Meier curve and Log-rank test. In the study, statistical significance was set at P < 0.05 (two-tailed).

## Results

### Serum IGFBP3 levels in EJA patients and normal controls

**Fig. 1:**
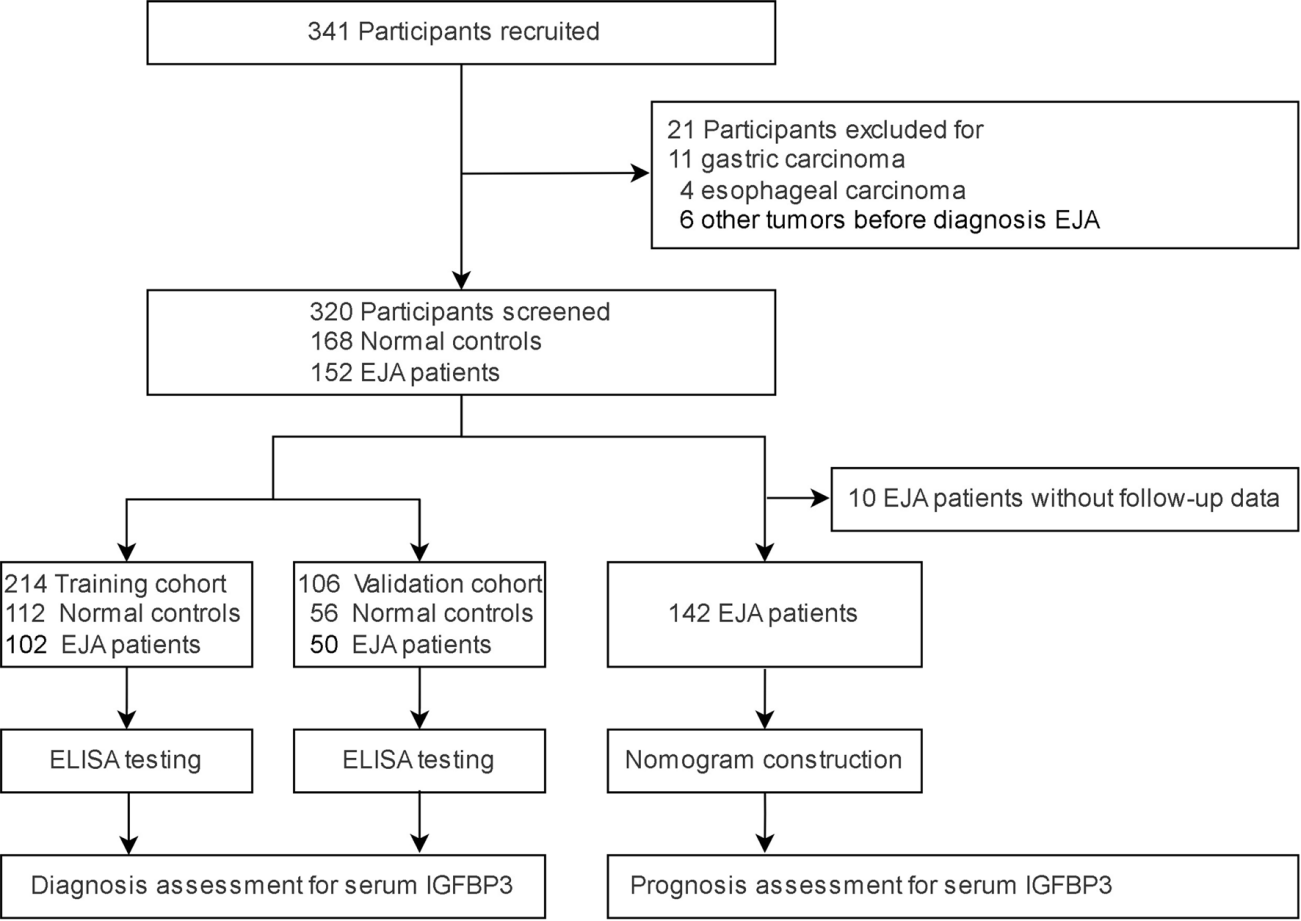
The study flowchart of serum IGFBP3 in EJA, showing steps involved in diagnosis and prognosis assessment for serum IGFBP3. *EJA* esophagogastric junction adenocarcinoma

**Fig. 2:**
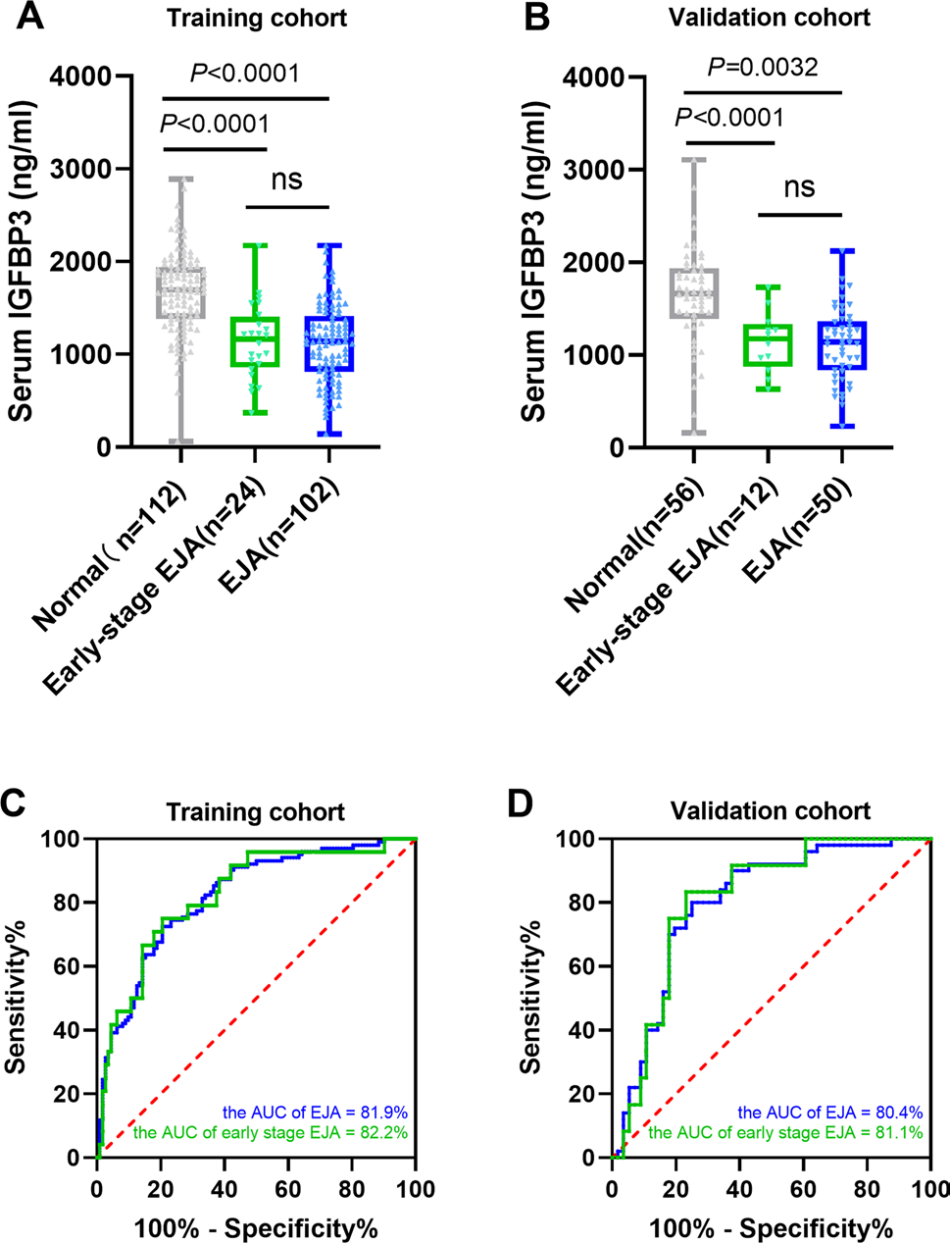
Level and ROC curve analysis of serum IGFBP3 for EJA. Serum IGFBP3 levels in normal controls, EJA and early-stage EJA patients in training cohort (**A**) and validation cohort (**B**) are shown in scatter plot and box plot. The lines in the box are means. The ROC curves of serum IGFBP3 to distinguish EJA and early-stage EJA patients and normal controls in training cohort (**C**) and validation cohort (**D**). The “EJA” curve (blue line) shows healthy subjects vs. all EJA subjects (early + late-stage), and the “early-stage EJA” curve (green line) shows healthy subjects vs. only the early-stage EJA subjects. *EJA* esophagogastric junction adenocarcinoma, *ROC* receiver-operating characteristics curve

We explored the expression of IGFBP3 in serum in EJA patients and normal controls. The flowchart for selection procedure of participants is shown in Fig. 1. A total of 320 participants were recruited and randomly divided into training cohort and validation cohort. There were 214 participants in the training cohort, including 102 EJA patients (24 early-stage EJA patients) and 112 normal controls. Correspondingly, the validation cohort of 106 participants included 50 EJA patients (12 early-stage EJA patients) and 56 normal controls. The clinical characters of the training cohort and validation cohort are shown in Table 1. Compared with the normal control group, serum IGFBP3 level of early-stage EJA or EJA patients in the training cohort were significantly lower (1664 ng/ml vs. 1138 ng/ml or 1121 ng/ml, P < 0.0001, Fig. 2A). The results in validation cohort were consistent (1632 ng/ml vs. 1142 ng/ml or 1124 ng/ml, P < 0.005, Fig. 2B).

**Table 1:** Clinical characteristics of samples in two cohorts

Variables	Training cohort				Validation cohort			
	EJA (n = 102)		Normal (n = 112)		EJA (n = 50)		Normal (n = 56)	
	No	%	No	%	No	%	No	%
Age (years)								
Mean ± SD	65 ± 8		56 ± 10		66 ± 7		53 ± 11	
Range	33–81		36–82		48–80		38–83	
Gender								
Female	22	21.6	44	39.3	8	16.0	23	41.1
Male	80	78.4	62	55.4	42	84.0	31	55.4
Unknown			6	5.4			2	3.6
Smoke								
No	42	41.2			24	48.0		
Yes	60	58.8			26	52.0		
Alcohol drinking								
No	78	76.5			35	70.0		
Yes	24	23.5			15	30.0		
Tumor size (cm)								
≤ 5	62	60.8			21	42.0		
> 5	40	39.2			29	58.0		
Tumor invasion depth								
T1 + T2 + T3	42	41.2			17	34.0		
T4	60	58.8			33	66.0		
Regional lymph nodes								
N0 + N1	58	56.9			23	46.0		
N2 + N3	44	43.1			27	54.0		
TNM stage								
Early stage (I + II)	24	23.5			12	24.0		
Advanced stage (III + IV)	78	76.5			38	76.0		
Serum IGFBP3 (ng/ml)								
Lower (≤ 1090 ng/ml)	42	41.2			20	40.0		
Higher (> 1090 ng/ml)	60	58.8			30	60.0		

*EJA* esophagogastric junction adenocarcinoma, *TNM* tumor node metastasis

### Diagnostic performance of serum IGFBP3 for EJA

With the use of ROC curve analysis, the AUC of serum IGFBP3 to distinguish EJA from normal controls was 0.819 (95% CI 0.763–0.875), and the sensitivity was 43.14% when the specificity was 90.18% in training cohort (Fig. 2C). Similar data were observed in validation cohort. With the same cut-off value of 1090 ng/ml from the training cohort, the AUC of serum IGFBP3 was 0.804 (95% CI 0.718–0.890), and the specificity and sensitivity were 87.50% and 42.00%, respectively, in validation cohort (Fig. 2D). Besides, serum IGFBP3 had a good diagnostic value for early-stage EJA (0.822, 90.18%, 45.83% and 0.811, 84.48%, 50.00% in training and validation cohorts, respectively). The predictive value and the likelihood ratios of serum IGFBP3 for improving the clinical interpretation of the diagnosis of early-stage EJA and EJA are also shown in Table 2. The analysis results showed that serum IGFBP3 had diagnostic value in differentiating early-stage EJA and EJA from normal controls.

**Table 2:** Diagnostic results of serum IGFBP3 in EJA

Group	AUC (95% CI)	Specificity (%)	Sensitivity (%)	NPV (%)	PPV (%)	NLR	PLR
Training cohort							
EJA vs. normal controls	0.819 (0.763–0.875)	90.18	43.14	63.52	80.00	0.63	4.39
Early-stage EJA vs. normal controls	0.822 (0.730–0.914)	90.18	45.83	88.60	50.00	0.60	4.67
Validation cohort							
EJA vs. normal controls	0.804 (0.718–0.890)	87.50	42.00	62.82	75.00	0.66	3.36
Early-stage EJA vs. normal controls	0.811 (0.696–0.926)	84.48	50.00	90.74	35.71	0.59	3.22

*EJA* esophagogastric junction adenocarcinoma, *AUC* area under the curve, *95% CI* 95% confidence interval, *NPV* negative predictive value, *PPV* positive predictive value, *NLR* negative likelihood ratio, *PLR* positive likelihood ratio

### Prognostic value of serum IGFBP3 for EJA

**Fig. 3:**
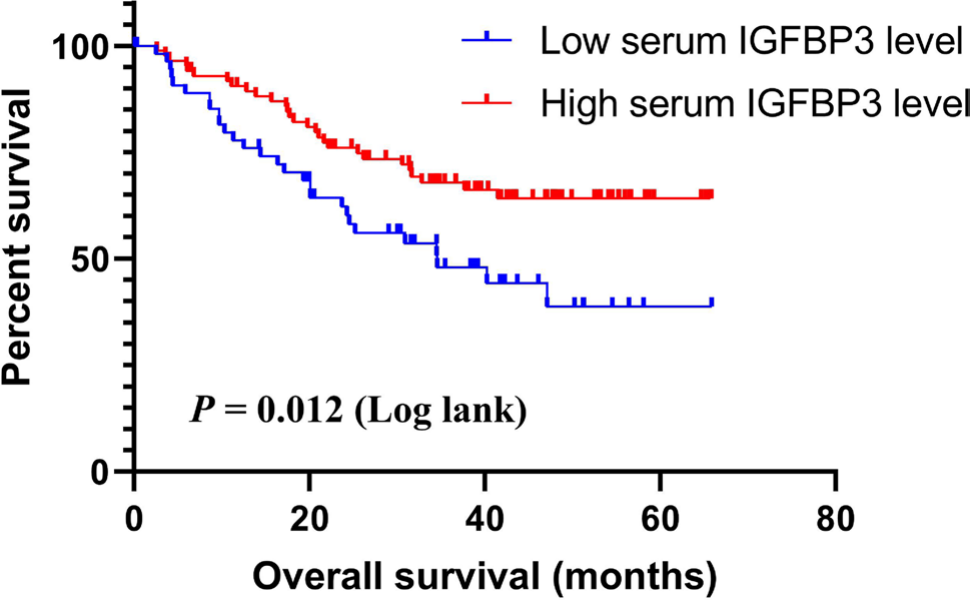
Kaplan–Meier curves for serum IGFBP3 with EJA patients. Patients with low serum IGFBP3 level had short survival. *EJA* esophagogastric junction adenocarcinoma

After finding that serum IGFBP3 had a good diagnostic ability in EJA, we further explored whether serum IGFBP3 could be applied for predicting prognosis. In our study, a total of 142 eligible EJA patients were analyzed. The cut-off value of serum IGFBP3 defined by X-tile was 1000 ng/ml. The median follow-up time was 31.62 months (interquartile range (IQR): 28.54–34.32 months). The median age was 66 years (IQR: 64–67 years). We made cox proportional hazards regression analysis of OS. The univariate analysis indicated that tumor size, tumor invasion depth, regional lymph nodes, TNM stage and serum IGFBP3 were associated with OS of EJA patients (P < 0.05). They were included in the multivariate analysis of OS. As shown in Table 3, the following variables remained independently prognostic: serum IGFBP3 (P = 0.005, HR = 0.468; 95% CI 0.275–0.794), tumor size (P = 0.018, HR = 1.993; 95% CI 1.126–3.526) and TNM stage (P = 0.014, HR = 2.010; 95% CI 1.151–3.510). Furthermore, Kaplan–Meier and log-rank test also revealed that EJA patients with lower serum IGFBP3 level had worse prognosis, compared with those with higher serum IGFBP3 level (P = 0.012, Fig. 3). These results indicated that there was prognostic value of IGFBP3 in EJA patients.

**Table 3:** Cox proportional hazards regression analysis of OS in EJA

Variables	Univariate analyses		Multivariate analyses	
	HR (95% CI)	*P-*value	HR (95% CI)	*P-*value
Age (years)				
≤ 64	Reference			
> 64	1.601 (0.925–2.770)	0.092		
Gender				
Female	Reference			
Male	1.274 (0.643–2.526)	0.488		
Smoke				
No	Reference			
Yes	1.270 (0.739–2.182)	0.387		
Alcohol drinking				
No	Reference			
Yes	1.311 (0.734–2.342)	0.360		
Tumor size (cm)			1.993 (1.126–3.526)	0.018
≤ 5	Reference			
> 5	2.304 (1.346–3.943)	0.002		
Tumor invasion depth				
T1 + T2 + T3	Reference			
T4	3.094 (1.598–3.990)	0.001		
Regional lymph nodes				
N0 + N1	Reference			
N2 + N3	3.130 (1.765–5.552)	0.001		
TNM stage			2.010 (1.151–3.510)	0.014
I–III	Reference			
IV	2.475 (1.460–4.197)	0.001		
Serum IGFBP3 (ng/ml)			0.468 (0.275–0.794)	0.005
Lower (≤ 1000 ng/ml)	Reference			
Higher (> 1000 ng/ml)	0.516 (0.305–0.873)	0.014		

*OS* overall survival, *EJA* esophagogastric junction adenocarcinoma, *HR* hazard ratio, *95% CI* 95% confidence interval, *TNM* tumor node metastasis

### Construction and performance evaluation of the nomogram

**Fig. 4:**
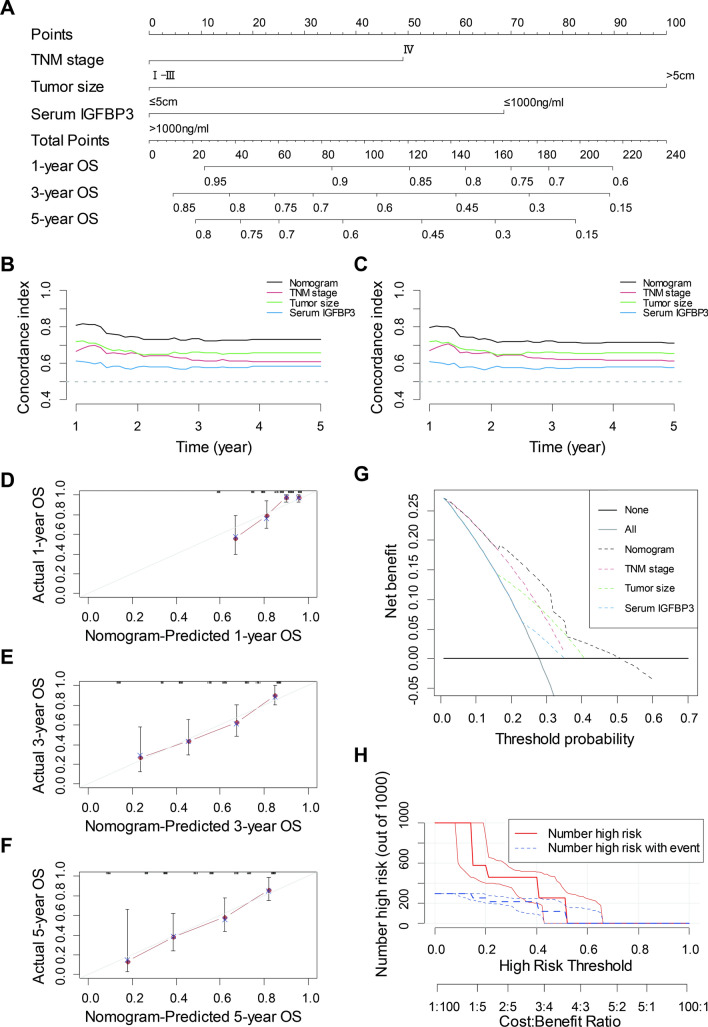
Construction and performance evaluation of the nomogram. Nomogram based on serum IGFBP3, tumor size and TNM stage to predict the 1-, 3- and 5-years OS for EJA patients. The nomogram used by adding the points determined on the point scale of each variable, the total points projected on the bottom scales match the probability of 1-, 3-, and 5-year OS of EJA patient (**A**). Time-dependent C-index of nomogram based on serum IGFBP3, tumor size and TNM stage for OS of EJA patient (**B**) and internally validated with using a bootstrap resampling method (**C**). Calibration plots showed good agreement for the survival probability at 1-, 3- and 5-year between the nomogram prediction and actual observed results (**D**–**F**). Decision curve analysis of serum IGFBP3, tumor size and TNM stage and nomogram. The straight black line represents the assumption that all patients die, and the horizontal line represents the assumption that no deaths happen (**G**). Clinical impact curve of nomogram. The red curve (number of high-risk individuals) indicated the number of people who were classifed as positive (high risk) by the nomogram at each threshold probability; the blue dotted curve (number of high-risk individuals with event) was the number of true positives at each threshold probability (**H**). *EJA* esophagogastric junction adenocarcinoma, *OS* overall survival

Using these selected independent prognostic markers containing serum IGFBP3, the nomogram was constructed for OS prediction (Fig. 4A). With a larger total point score, the OS was shorter. A nomogram was used by adding the points determined on the point scale of each variable. The total points projected on the bottom scales showed the probability of 1-, 3-, and 5-year survival. For example, a patient, regardless of age or sex, with TNM I stage, Tumor size > 5 cm, and serum IGFBP3 ≤ 1000 ng/ml had a total of 168 points indicating an estimated 1-, 3-, and 5-y OS of 73%, 30%, and 22%, respectively.

We used the C-index to evaluate the discrimination of the prognostic nomogram, which enumerated the level of consistency between the predicted and observed OS. The C-index based on the new nomogram (0.735, 95% CI 0.673–0.797) for OS in the cohort was much higher than that of the TNM stage (0.625, 95% CI 0.562–0.689, P = 0.001, Table 4). The nomogram exhibited better discrimination than single prognostic marker to predict OS (Fig. 4B). And consistent result was also observed in internal validation using a bootstrap resampling method (Fig. 4C). Then we used the calibration plots to assess the calibration of the prognostic nomogram, and the plot showed the prediction of the nomogram at 1-, 3-, and 5-year basically matched with the actual observation (Fig. 4D–F). Moreover, it was noted that the AIC and BIC of the new nomogram for OS were much lower than those of TNM stage (486.941 vs. 506.171; 493.017 vs. 508.197, respectively), indicating that the new nomogram had a higher goodness-of-fit in predicting the OS of EJA patients.

**Table 4:** The C-index, AIC and BIC of prognostic factors and nomogram for prediction OS

Factors	C-index (95% CI)	*P-*value	AIC	BIC
Serum IGFBP3	0.579 (0.513–0.645)		511.279	513.304
Tumor size	0.658 (0.600–0.717)		496.006	498.031
TNM stage	0.625 (0.562–0.689)		506.171	508.197
Nomogram	0.735 (0.673–0.797)		486.941	493.017
Nomogram vs. Serum IGFBP3		< 0.001		
Nomogram vs. Tumor size		0.014		
Nomogram vs. TNM stage		0.001		

*C-index* concordance index, *AIC* Akaike Information Criterion, *BIC* Bayesian Information Criterion, *OS* overall survival, *95% CI* 95% confidence interval, *TNM* tumor node metastasis, *Nomogram* serum IGFBP3 + Tumor size + TNM stage

Net benefit and predictive capacity of the nomogram were displayed by DCA, of which represented the nomogram (dark dotted line) had a higher net benefit than that of TNM staging (red dotted line) or other risk indicators to predict OS (Fig. 4G). Both IDI and NRI suggested that the predictive accuracy of the nomogram had improvements when predicting 1-, 3-, and 5-year OS (Table 5). As shown in Fig. 4H, the CIC intuitively showed that nomogram had high clinical net benefit, and confirmed the clinical value of prognosis nomogram. To sum up, compared with the traditional TNM staging evaluation system, the new nomogram model containing IGFBP3 had more excellent performance in terms of prognosis prediction for EJA patients.

**Table 5:** Predictive improvement of the nomogram

	1-year				3-year				5-year			
	NRI %	*P*-value	IDI %	*P-*value	NRI %	*P-*value	IDI %	*P-*value	NRI %	*P-*value	IDI %	*P-*value
OS												
Nomogram vs. Serum IGFBP3	47.3	0.002	11.9	< 0.001	38.5	0.002	16.8	0.002	31.1	0.154	13.9	0.138
Nomogram vs. Tumor size	24.1	0.054	6.6	0.018	20.9	0.034	7.1	0.046	14.0	0.402	8.2	0.120
Nomogram vs. TNM stage	27.7	0.016	9.3	0.002	26.0	0.014	13.9	0.004	31.1	0.180	18.0	0.014

*NRI* net reclassification improvement, *IDI* integrated discrimination improvement, *OS* overall survival, *Nomogram* serum IGFBP3 + Tumor size + TNM stage, *TNM* tumor node metastasis

### Risk stratification based on the nomogram

**Fig. 5:**
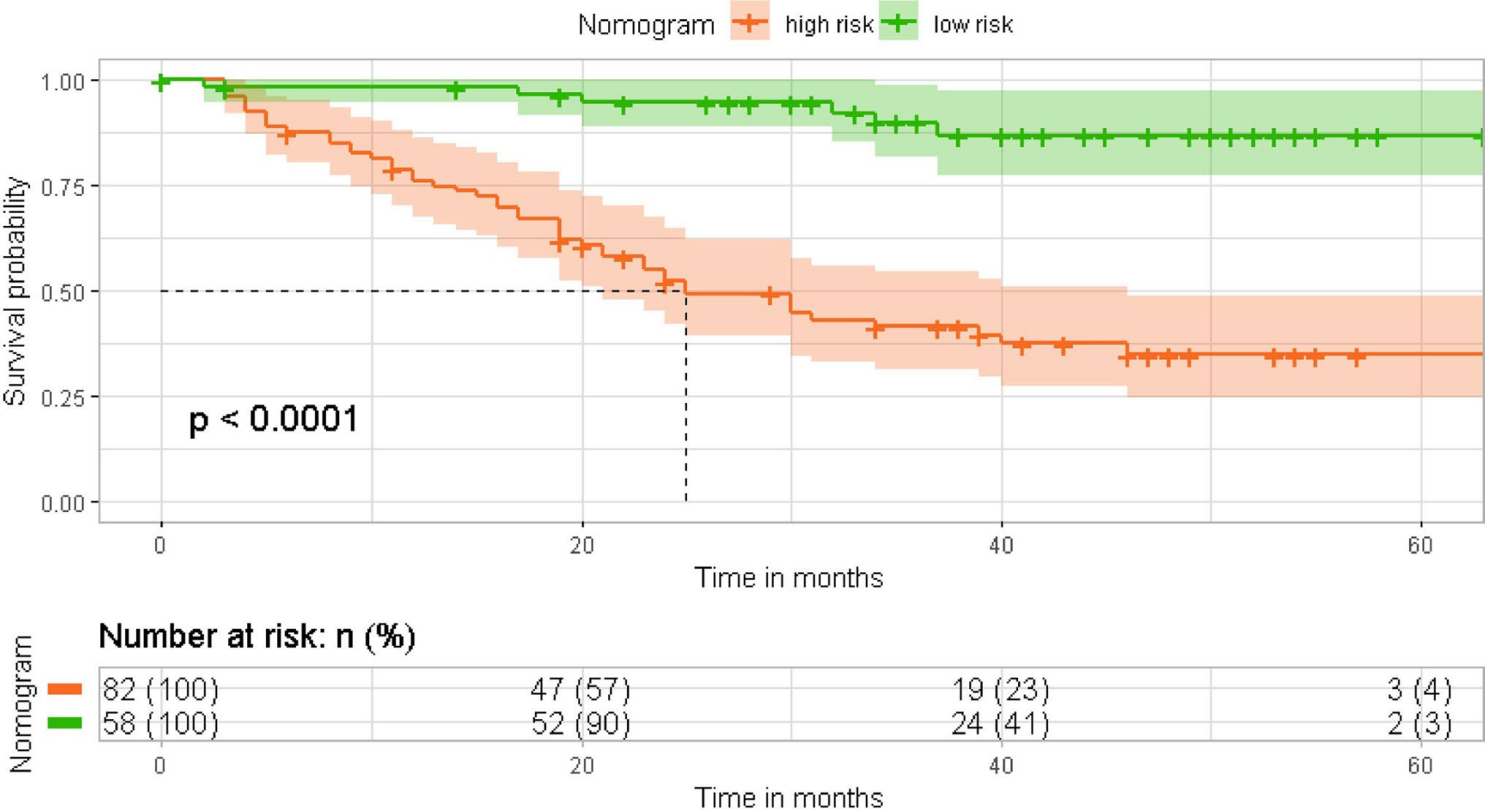
Survival curve of risk stratification for OS based on prediction of nomogram. Patients in high-risk group had short survival. Low risk: total points ≤ 100. High risk: total points > 100. *OS* overall survival

In order to verify whether the newly constructed nomogram can effectively stratify risks in EJA patient, we calculated the predicted total points based on the nomogram and used X-tile software to find the best cut-off value for OS (100.00), and then subdivided EJA patients into low- and high-risk groups. The Kaplan–Meier survival analysis was applied to assess their survival. As anticipated, compared with the low-risk group, the OS of the high-risk group was significantly shorter (P < 0.0001, Fig. 5). The result demonstrated that the nomogram could effectively separate OS for the two proposed risk groups.

### Pertinent bioinformatic analyses of IGFBP3

Above, we studied the value of serum IGFBP3 in the diagnosis and prognosis of EJA, using samples collected from the Cancer Hospital of Shantou University Medical College. Then we tried to further explore other potential values of IGFBP3 by using the public database. The Human Protein Atlas database is a very important and useful database, which can supplement clinical data. However, we found that human tissue samples of EJA were not available in this database. Thus, we analyzed the gastric cancer samples instead (esophageal cancer sample are also not available), and we found that IGFBP3 showed no prognostic value in gastric cancer (Additional file 1: Fig. S1). Whether the same result would be observed in EJA needs to be assessed in future study. Next, we used MethSurv (https://biit.cs.ut.ee/methsurv/) to analyze the effect of DNA methylation of IGFBP3. We presented heatmap and prognostic value of DNA methylation clustering the expression levels of the IGFBP3 gene in esophageal carcinoma (ESCA) and stomach adenocarcinoma (STAD) (Additional file 1: Fig. S2 and Tables S1–2). Results concluded that cg04690927 from IGFBP3 in ESCA and cg24381682 from IGFBP3 in STAD had the highest DNA methylation levels and significant prognostic value (likelihood ratio (LR) test P < 0.05).

## Discussion

For recent decades, as a kind of junctional cancer, the incidence of EJA has been on the rise, and its definition, evaluation, and management are still unclear. Until now, there has been no reports about serum IGFBP3 in diagnostic and prognosis of EJA. In our study, we found that serum IGFBP3 had good potential in diagnosis of EJA, with the AUC values of 0.819 and 0.804 in training and validation cohorts, respectively. In addition, low IGFBP3 level was associated with poor prognosis of EJA patients. Then, we developed and validated a new prognostic nomogram containing serum IGFBP3, tumor size and TNM stage for OS prediction of EJA patients. Importantly, compared with the traditional TNM stage system, the nomogram showed enhanced predictive accuracy and discriminative ability. It successfully stratified the risk of EJA patients and performed well in the internal validation.

IGFBP3, a member of IGFBP family, had been reported that it can halt cell proliferation, promote cell apoptosis and reduce tumor growth [31, 32]. The main function of IGFBP3 is to transport IGF-I and IGF-II in circulation, thus prolonging the half-life of IGFs. In addition, IGFBP3 can also regulate the availability and activity of IGF at the cellular level by autocrine or paracrine [33]. IGFBP3 can restrain or enhance the IGF actions, mainly depending on cell types, cell environment and IGFBP3 concentration [34]. Apart from age, other factors are known to affect serum IGFBP3 concentration, such as gender, exercise and high carbohydrate diet [35–37]. Besides, the IGF-IGFBP system is related to the onset and development of cancer [38].

Previous studies indicated that there might be potential clinical value of IGFBP3 in several forms of cancers [39, 40]. Vadgama et al. [41] found that breast cancer patients have elevated serum IGFBP3 levels. However, serum IGFBP3 levels in hepatocellular carcinoma patients were significantly lower than those in healthy individuals [42]. Similarly, Patients with lung cancer have lower serum IGFBP3 levels [43]. These results implied that the expression levels of serum IGFBP3 were different in various carcinomas. Moreover, it should be pointed out that all these studies lacked analysis of the diagnostic value of serum IGFBP3. Here we showed that serum IGFBP3 had potential value in the early diagnosis of EJA. For a useful diagnostic test, the sensitivity and specificity would be as high as possible. However, to qualify as a clinically useful biomarker for detection of tumor disease, it should have better test characteristics (sensitivity, specificity) than currently applied tumor markers. As far as we know, Carcinoembryonic antigen (CEA) and carbohydrate antigen 19-9 (CA19-9) have been used as major serum tumor markers in gastrointestinal cancers, of which the positive rates for EJA patients were only 20.3% and 12.9%, respectively [44]. Thus, serum IGFBP3 potentially demonstrated a better diagnostic sensitivity for EJA than CEA and CA19-9 currently used in clinical practice. On the other hand, the sensitivity of this test would be not high enough to be used for screening purposes in general or high-risk populations. It is notable that studies increasingly suggest that detection of combined serum biomarkers in an optimized panel would be of great significance for improving early diagnostic value. Thus, we believe that serum IGFBP3 might serve as a potential candidate biomarker for the construction of an optimized biomarker panel for early detection of EJA.

On the other hand, the value of serum IGFBP3 in tumor prognosis should not be ignored. Efficient and accurate prognosis prediction can help guide patients' health management, treatment and lifestyle intervention, and improve their quality of life. In practice, clinicians evaluate and manage EJA patients according to the traditional AJCC TNM classification [45]. However, TNM classification can't accurately judge the prognosis of EJA, because EJA is considered to have different biological characteristics compared with gastric cancer and esophageal cancer [46]. At present, molecular markers have attracted wide attention because of their increasingly important role in tumor prognosis. In this study, cox regression analysis showed that serum IGFBP3 was an independent prognostic factor of EJA, and its low expression was related to the poor prognosis of EJA patients. Similar result was reported that low serum IGFBP3 level may be related to poor prognosis of esophageal cancer, increased cancer risk and tumor metastasis [47]. To date, there are several nomograms for EJA patients, which are based on hematological indicators. Charles et al. [48] established a nomogram for patients with advanced gastric cancer or EJA who received drug treatment, including hematological indexes such as albumin and serum sodium. Chau et al. [49] constructed a prognosis model for patients with locally advanced or metastatic esophageal gastric cancer, which contains ALP as a serum enzymology index. However, these studies did not directly target EJA patients, and the sample size of EJA patients was small, which reduces the ability of these models to predict EJA prognosis. Our nomogram based on serum IGFBP3, tumor size and TNM stage improved the prognostic prediction of EJA with concordance index of 0.735. Compared with the traditional TNM staging evaluation system, both IDI and NRI suggested that the nomogram has a better performance in integrated discrimination and net reclassification improvement when predicting 1-, 3-, and 5-year OS.

Besides, numerous studies have validated that age was one of the key factors affecting serum IGFBP3 levels, and there were differences in serum IGFBP3 levels among different age groups [50]. However, in our study, there was no significant statistical difference between serum IGFBP3 level and age. This should be verified in future studies. In addition, it was worth noting that the serum IGFBP3 level in our control subjects was lower than that of the normal control group reported in other literatures [50, 51]. Different levels of serum IGFBP3 in normal controls observed in these studies might be due to discrepancies in race, diet and geography. For example, it was reported diet could affect the serum concentration of IGFBP3 [52]. Moreover, different testing methods and manufacturers may also cause inconsistent results.

Although our results evaluating serum IGFBP3 for the diagnostic and prognosis of EJA are promising, there were some limitations in this study: First, our validation samples were from the same hospital, and a large-scale multi-center verification is needed in the future. Second, the number of early-stage EJA patients enrolled in this study was relatively small. It's a fact that the vast majority of EJA patients are diagnosed in advanced stages, thus resulting in the difficult to obtain a large sample size of early-stage patients in clinical practice. Despite the above-mentioned shortcomings, we are confident that our findings will help elucidate the value of serum IGFBP3 in the diagnosis and prognosis of EJA patients.

In summary, our result demonstrated serum IGFBP3 could be used as a potential biomarker for early diagnosis. Moreover, serum IGFBP3 was identified as an independent prognostic factor of EJA, and its low expression was related to the poor prognosis of EJA patients. A multi-parametric prognostic nomogram, which was composed of serum IGFBP3, tumor size and TNM stage, showed improved performance in individualized prognosis estimation compared with the traditional TNM staging. In the future work, multi-center verification with large early-stage patients is needed to validate the value of serum IGFBP3 assays in early diagnosis and prognostic prediction of EJA.

## Supplementary Information

**Additional file 1: Fig. S1.** IGFBP3 in stomach cancer by The Human Protein Atlas database. Results concluded that IGFBP3 is not prognostic in stomach cancer. **Fig. S2.** Heatmap of DNA methylation expression levels of the IGFBP3 gene in ESCA and STAD by MethSurv platform. cg00419512, cg05867388, cg06789764 of IGFBP3 displays the highest level of DNA methylation in ESCA (A). cg00419512, cg05867388, cg06789764 of IGFBP3 displays the highest level of DNA methylation in STAD (B). ESCA, esophageal carcinoma; STAD, Stomach adenocarcinoma. **Table S1.** Prognostic Value of Single CpG of the IGFBP3 gene in ESCA by MethSurv platform. **Table S2.** Prognostic Value of Single CpG of the IGFBP3 gene in STAD by MethSurv platform

## Data Availability

The datasets used and analyzed during the current study are available from the corresponding author on reasonable request.

## Code availability

Statistical analyses were performed using SPSS software (version 26.0, IBM Corp., Chicago, IL, USA), GraphPad Prism software (version 8.0.2, http://www.graphpad.com) and R (version 4.2.1, http://www.R-project.org) for Windows.
