# Supplementary material for: Serum insulin-like growth factor binding protein 3 as a promising diagnostic and prognostic biomarker in esophagogastric junction adenocarcinoma
Source: Discov Oncol. 2022 Nov 21;13:128. doi: 10.1007/s12672-022-00591-1 (PMC9679126; doi:10.1007/s12672-022-00591-1)
Supplement: Supplementary file 1 — Additional file 1: Fig. S1. IGFBP3 in stomach cancer by The Human Protein Atlas database. Results concluded that IGFBP3 is not prognostic in stomach cancer. Fig. S2. Heatmap of DNA methylation expression levels of the IGFBP3 gene in ESCA and STAD by MethSurv platform. cg00419512, cg05867388, cg06789764 of IGFBP3 displays the highest level of DNA methylation in ESCA (A). cg00419512, cg05867388, cg06789764 of IGFBP3 displays the highest level of DNA methylation in STAD (B). ESCA, esophageal carcinoma; STAD, Stomach adenocarcinoma. Table S1. Prognostic Value of Single CpG of the IGFBP3 gene in ESCA by MethSurv platform. Table S2. Prognostic Value of Single CpG of the IGFBP3 gene in STAD by MethSurv platform [file 12672_2022_591_MOESM1_ESM.pdf]

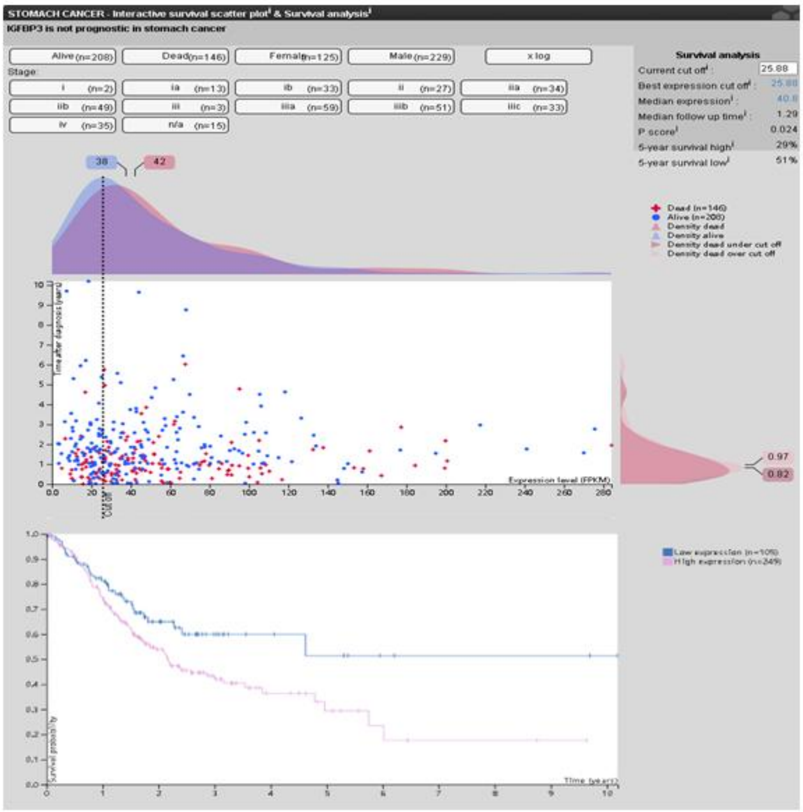

Fig. S1 IGFBP3 in stomach cancer by The Human Protein Atlas database. Results concluded that IGFBP3 is not prognostic in stomach cancer

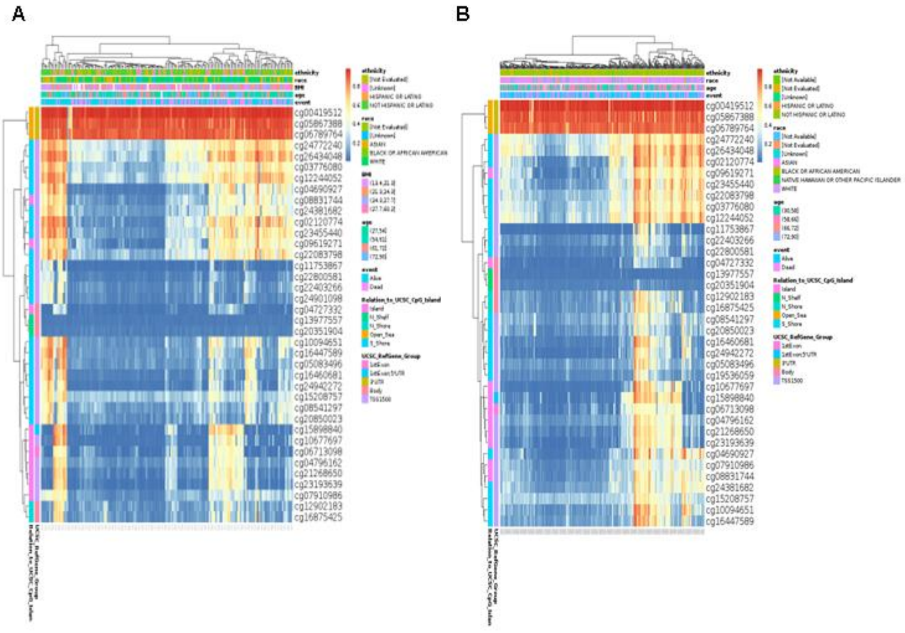

Fig. S2 Heatmap of DNA methylation expression levels of the IGFBP3 gene in ESCA and STAD by MethSurv platform. cg00419512, cg05867388, cg06789764 of IGFBP3 displays the highest level of DNA methylation in ESCA (A). cg00419512, cg05867388, cg06789764 of IGFBP3 displays the highest level of DNA methylation in STAD (B). ESCA, esophageal carcinoma; STAD, Stomach adenocarcinoma

Table S1 Prognostic Value of Single CpG of the IGFBP3 gene in ESCA by MethSurv platform

Gene-CpG	Cancer	HR	LR_test_pvalue
IGFBP3-TSS1500-S_Shore-cg04690927	ESCA	1.921	0.019*
IGFBP3-TSS1500-S_Shore-cg24381682	ESCA	1.853	0.024*
IGFBP3-TSS1500-Island-cg08831744	ESCA	1.707	0.043*
IGFBP3-TSS1500-Island-cg23193639	ESCA	1.598	0.043*
IGFBP3-TSS1500-S_Shore-cg22083798	ESCA	1.581	0.091
IGFBP3-TSS1500-S_Shore-cg02120774	ESCA	1.538	0.120
IGFBP3-TSS1500-Island-cg21268650	ESCA	1.533	0.109
IGFBP3-TSS1500-Island-cg07910986	ESCA	1.449	0.106
IGFBP3-TSS1500-Island-cg09619271	ESCA	1.447	0.108
IGFBP3-TSS1500-S_Shore-cg26434048	ESCA	1.444	0.170
IGFBP3-1stExon;5'UTR-Island-cg15898840	ESCA	1.435	0.120
IGFBP3-TSS1500-S_Shore-cg23455440	ESCA	1.417	0.202
IGFBP3-TSS1500-S_Shore-cg12244052	ESCA	1.416	0.187
IGFBP3-TSS1500-Island-cg04796162	ESCA	1.402	0.143
IGFBP3-TSS1500-Island-cg10677697	ESCA	1.396	0.146
IGFBP3-TSS1500-S_Shore-cg22800581	ESCA	1.396	0.198
IGFBP3-TSS1500-S_Shore-cg05083496	ESCA	1.324	0.296
IGFBP3-1stExon-Island-cg06713098	ESCA	1.308	0.269
IGFBP3-TSS1500-S_Shore-cg03776080	ESCA	1.304	0.317
IGFBP3-TSS1500-S_Shore-cg10094651	ESCA	1.271	0.294
IGFBP3-Body-N_Shelf-cg20351904	ESCA	1.234	0.358
IGFBP3-TSS1500-S_Shore-cg24772240	ESCA	1.209	0.475
IGFBP3-Body-Island-cg04727332	ESCA	1.204	0.423
IGFBP3-TSS1500-S_Shore-cg15208757	ESCA	0.902	0.654
IGFBP3-TSS1500-S_Shore-cg24942272	ESCA	0.874	0.609
IGFBP3-3'UTR-Open_Sea-cg05867388	ESCA	0.855	0.540
IGFBP3-Body-N_Shore-cg16875425	ESCA	0.852	0.537
IGFBP3-TSS1500-S_Shore-cg16460681	ESCA	0.843	0.540
IGFBP3-3'UTR-Open_Sea-cg06789764	ESCA	0.76	0.276
IGFBP3-TSS1500-S_Shore-cg16447589	ESCA	0.722	0.270
IGFBP3-TSS1500-S_Shore-cg24901098	ESCA	0.72	0.159
IGFBP3-TSS1500-S_Shore-cg08541297	ESCA	0.715	0.148
IGFBP3-Body-N_Shelf-cg13977557	ESCA	0.714	0.190
IGFBP3-TSS1500-S_Shore-cg22403266	ESCA	0.69	0.109
IGFBP3-TSS1500-S_Shore-cg20850023	ESCA	0.674	0.148
IGFBP3-TSS1500-S_Shore-cg11753867	ESCA	0.666	0.082
IGFBP3-3'UTR-Open_Sea-cg00419512	ESCA	0.621	0.039*
IGFBP3-Body-N_Shore-cg12902183	ESCA	0.583	0.038*

ESCA, esophageal carcinoma. * indicates signifcant diference

Table S2 Prognostic Value of Single CpG of the IGFBP3 gene in STAD by MethSurv platform

Gene-CpG	Cancer	HR	LR_test_pvalue
IGFBP3-Body-N_Shelf-cg13977557	STAD	1.245	0.181
IGFBP3-Body-N_Shelf-cg20351904	STAD	0.862	0.365
IGFBP3-3'UTR-Open_Sea-cg05867388	STAD	0.784	0.189
IGFBP3-3'UTR-Open_Sea-cg06789764	STAD	0.783	0.210
IGFBP3-TSS1500-S_Shore-cg26434048	STAD	0.778	0.124
IGFBP3-TSS1500-S_Shore-cg24772240	STAD	0.774	0.171
IGFBP3-TSS1500-S_Shore-cg04690927	STAD	0.755	0.087
IGFBP3-TSS1500-S_Shore-cg22800581	STAD	0.75	0.139
IGFBP3-TSS1500-S_Shore-cg22083798	STAD	0.738	0.070
IGFBP3-TSS1500-S_Shore-cg23455440	STAD	0.738	0.101
IGFBP3-TSS1500-Island-cg08831744	STAD	0.731	0.065
IGFBP3-TSS1500-S_Shore-cg03776080	STAD	0.728	0.053
IGFBP3-TSS1500-Island-cg23193639	STAD	0.726	0.074
IGFBP3-TSS1500-S_Shore-cg24381682	STAD	0.723	0.048*
IGFBP3-TSS1500-S_Shore-cg19536059	STAD	0.718	0.091
IGFBP3-TSS1500-S_Shore-cg08541297	STAD	0.715	0.054
IGFBP3-TSS1500-Island-cg07910986	STAD	0.714	0.040*
IGFBP3-Body-N_Shore-cg12902183	STAD	0.711	0.063
IGFBP3-TSS1500-S_Shore-cg02120774	STAD	0.705	0.033*
IGFBP3-TSS1500-S_Shore-cg16447589	STAD	0.697	0.053
IGFBP3-TSS1500-Island-cg09619271	STAD	0.683	0.020*
IGFBP3-TSS1500-S_Shore-cg15208757	STAD	0.681	0.047*
IGFBP3-TSS1500-S_Shore-cg05083496	STAD	0.68	0.048*
IGFBP3-TSS1500-S_Shore-cg12244052	STAD	0.673	0.042*
IGFBP3-TSS1500-Island-cg10677697	STAD	0.67	0.036*
IGFBP3-TSS1500-S_Shore-cg11753867	STAD	0.668	0.040*
IGFBP3-TSS1500-Island-cg04796162	STAD	0.664	0.013*
IGFBP3-3'UTR-Open_Sea-cg00419512	STAD	0.659	0.042*
IGFBP3-1stExon;5'UTR-Island-cg15898840	STAD	0.659	0.036*
IGFBP3-Body-N_Shore-cg16875425	STAD	0.659	0.038*
IGFBP3-TSS1500-Island-cg21268650	STAD	0.648	0.027*
IGFBP3-1stExon-Island-cg06713098	STAD	0.633	0.021*
IGFBP3-TSS1500-S_Shore-cg20850023	STAD	0.617	0.015*
IGFBP3-TSS1500-S_Shore-cg22403266	STAD	0.608	0.014*
IGFBP3-Body-Island-cg04727332	STAD	0.603	0.002**
IGFBP3-TSS1500-S_Shore-cg10094651	STAD	0.602	0.010**
IGFBP3-TSS1500-S_Shore-cg24942272	STAD	0.596	0.009**
IGFBP3-TSS1500-S_Shore-cg16460681	STAD	0.595	0.009**

STAD, Stomach adenocarcinoma. * indicates signifcant diference
